# Trajectories of persisting Covid- 19 symptoms up to 24 months after acute infection: findings from the Predi-Covid cohort study

**DOI:** 10.1186/s12879-025-11023-0

**Published:** 2025-04-25

**Authors:** Aurélie Fischer, Lu Zhang, Abir Elbéji, Paul Wilmes, Chantal J. Snoeck, Jérôme Larché, Pauline Oustric, Markus Ollert, Guy Fagherazzi

**Affiliations:** 1 Department of Precision Health, Deep Digital Phenotyping Research Unit, Luxembourg Institute of Health, 1 A-B Rue Thomas Edison, Strassen, L- 1445 Luxembourg; 2 Université de Lorraine, Nancy, France; 3 Bioinformatics Platform, Luxembourg Institute of Health, 1 A-B, Rue Thomas Edison, Strassen, L- 1445 Luxembourg; 4 Luxembourg Centre for Systems Biomedicine (LCSB), University of Luxembourg, Campus 20 Belval, 7, Avenue Des Hauts-Fourneaux, Esch-Sur-Alzette, L- 4362 Luxembourg; 5 Department of Life Sciences and Medicine, Faculty of Science, Technology and Medicine, 7, Avenue Des Hauts-Fourneaux, Esch-Sur-Alzette, L- 4362 Luxembourg; 6 Department of Infection and Immunity, Clinical and Applied Virology Group, Luxembourg Institute of Health, 29, Rue Henri Koch, Esch-Sur-Alzette, L- 4354 Luxembourg; 7 Long Covid Center, Clinique du Parc, Castelnau-Le-Lez, France; 8 Association #ApresJ20 Covid Long France, Lucé, France; 9 Department of Infection and Immunity, Luxembourg Institute of Health, 29, Rue Henri Koch, Esch-Sur-Alzette, L- 4354 Luxembourg

**Keywords:** COVID- 19, SARS-COV- 2, Long Covid symptoms, Trajectories, Latent class mixed models, Quality of life

## Abstract

**Introduction:**

Long COVID is a multisystemic, fluctuating condition inducing a high burden on affected people. Despite the existence of some guidelines, its management remains complicated. We aimed to demonstrate that symptoms after a COVID-19 infection evolve following different trajectories from the initial infection until 24 months after, to identify the determinants of these trajectories, and the quality of life of people in these trajectories.

**Methods:**

Study participants from the Predi-COVID cohort were digitally followed from their acute SARS-CoV-2 infection until a maximum of 24 months. Data from 10 common symptoms collected at study inclusion, and months 12, 15, and 24 awere used to create a total symptom score. Impact of symptoms on quality of life was assessed at month 24 using standardized questionnaires and ad-hoc questions. Latent classes mixed models were used to identify total score symptom trajectories and individual symptoms trajectories.

**Results:**

We included 555 participants with at least 2 different time points available during follow-up (Baseline and at least one of the M12, M15 or M24 questionnaires). We identified 2 total symptom score trajectories: T1 “Mild symptoms, fast resolution” (*N* = 376; 67.7%), and T2 “Elevated and persisting symptoms” (*N* = 179; 32.3%). The main determinants of being in T2 were: older age (OR = 1.86; *p* = 0.003), to be a woman (OR = 1.81; *p* = 0.001)), elevated BMI (OR = 3.97; *p* < 0.001), and the presence of multi comorbidities (OR = 2.67; *p* = 0.005). Symptoms impacted the quality of life more in T2 than in T1 at 24 months (high fatigue level: 64.8% vs 19.5%, altered respiratory quality of life: 42.6% vs 4.6%, anxiety: 24.1% vs 4.6%, stress: 57.4% vs 35.6%, and bad sleep: 75.9% vs 51.1%).

**Conclusion:**

A third of our study population was in the T2 “Elevated and persisting symptoms” trajectory, presenting high symptom frequencies up to 24 months after initial infection, with a significant impact on quality of life. This work underlined the urgent need to better identify individuals most vulnerable to long-term complications to develop tailored interventions for them.

**Trial registration:**

Clinicaltrials.gov NCT04380987 (date of registration: 2020–05-07).

**Supplementary Information:**

The online version contains supplementary material available at 10.1186/s12879-025-11023-0.

## Background

Four years since the pandemic started, it has been estimated that more than 65 millions of people are still suffering from long-term sequelae grouped under the term Long COVID or Post COVID which became a major public health issue worldwide [1].

Long COVID definition evolved with time and the latest one has been defined in July 2024 by the “National Academies of Sciences, Engineering, and Medicine Committee on Examining the Working Definition for Long Covid” (NASEM and states that “Long Covid is an infection-associated chronic condition that occurs after SARS-CoV- 2 infection and is present for at least 3 months as a continuous, relapsing and remitting, or progressive disease state that affects one or more organ systems” [2]. It has been estimated that 10–20% of people infected by SARS-CoV- 2 develop Long COVID. All age categories are concerned and people with mild acute illness represent a majority of them.

Long COVID is characterized by a large variety of symptoms, affecting many organs and has a high impact on the quality of life of affected people [3]. In addition, the impact of Long COVID could be evidenced by an increased disability-adjusted life years (DALYs) and mortality rate at 1, 2, and 3 years after initial infection, with a trend to improvement at 3 years [4]. Long COVID can also lead to the onset of new comorbidities like diabetes [5].

The economic impact of Long COVID is also important with a varying number of people with Long COVID that had to stop working, reduce their working time, or retire earlier than foreseen, depending on the country [6, 7]. In the US, the annual total cost of Long COVID taking into account the cost of reduced earnings, of medical spendings and of reduced quality of life, has been estimated around $3.7 trillion, representing 17% of the GDP [8].

In the absence of medical treatment, the management of Long COVID primarily involves the incorporation of various strategies that encompass symptom-specific care such as neurocognitive issues, physical rehabilitation for senses like taste and smell, along with dietary and activity adjustments. Some antiviral treatments are currently under clinical evaluation. In particular, some studies showed that early administration of Nilmatrelvir boosted with Ritonavir or Molnupiravir after COVID- 19 infection seemed to reduce the risk of Long COVID [9–11].

Pacing stands out as the primary recommendation for managing activities, emphasizing the importance of balancing exertion with rest to prevent worsening of symptoms [12]. Vaccination has been consistently shown by studies to be an effective prevention measure with a decrease of 15 to 75% of Long COVID risk, with an average risk reduction of around 40% [13–15].

Despite progressing knowledge about biological mechanisms, epidemiology, clinical manifestation, and risk factors, Long COVID care still faces many challenges and unmet needs [15].

Long COVID has also been shown to be heterogeneous [16], with a wide variety of symptoms [3], and affected people could be classified into different sub-groups of various Long COVID severity [17, 18]. Only a few studies reported long-term evolution (up to 24 months or more) [19–21] and it is crucial to better understand how and why some people with Long COVID evolve differently over time to help physicians to personalize the care of people with Long COVID.

In this study, we hypothesized that COVID- 19 symptoms evolved following different trajectories with a differential impact on the quality of life of affected people.

We thus aimed at 1) identifying symptom trajectories from the acute infection until 24 months after, among a cohort of initially SARS-CoV- 2 positive adults, 2) describing individual characteristics and identifying the main determinants of the trajectories, and 3) assessing multi-dimensions of the quality of life of people in the different trajectories.

## Methods

### Population and study design

In this study we analyzed the data from participants in the Predi-COVID study, a prospective cohort study of persons with a PCR-confirmed SARS-CoV- 2 infection in Luxembourg. The Predi-COVID study design and analysis plan has been published previously [22]. The study is registered in ClinicalTrials.gov (NCT04380987) and was approved by the National Research Ethics Committee of Luxembourg (study number 202003/07) in April 2020. All participants signed an informed consent before inclusion in the study. Inclusion criteria were to be an adult person with a PCR-confirmed SARS-CoV 2 infection in Luxembourg, hospitalized or not during acute infection.

Data were collected longitudinally, from baseline to a maximum of 24 months. Baseline data were collected by phone by an experienced clinical research nurse at study inclusion, which was done in the 5 days after the PCR test result and consisted of individual characteristics and symptoms. Participants were then invited to complete detailed self-reported questionnaires on symptoms and quality of life at months 12, 15 and 24 after inclusion in the study (full questionnaire provided in supplementary file, additional file 1).

### Study design

This study is a longitudinal analysis of participants'symptoms and health status from acute infection to a maximum of 24 months after. Participants included between May 1 st, 2020 and September 30 th, 2021, who provided the baseline data and completed at least one of the M12, M15 or M24 questionnaires were eligible for the present study (*N* = 555).

### Symptoms

We used a list of 10 symptoms (fatigue, cough, sore throat, diarrhea, chest pain, myalgia, shortness of breath, conjunctivitis, rash, and fever) collected at baseline, M12, M15, and M24. This list was elaborated and limited to the 10 symptoms common to baseline and all follow-up timepoints. Although many additional symptoms were collected at M12, 15 and 24, they were not collected at baseline due to limited knowledge of the disease available at the pandemic’s start, and could thus not be used to build trajectories starting at baseline. The question in the M12 - 15–24 questionnaire was formulated as follows: “Have you noticed the following symptoms or illnesses since your Covid- 19 diagnosis? “ and the response modalities were 1/“yes and I still feel it today”, 2/“yes, I had it but I no longer have it”, and 3/“no, I have never had this symptom”.

We considered response 1/as stating the presence of the symptom.

The addition of symptoms reported by the participants at each time point corresponds to the “total symptom score” variable.

### Covariates

The following covariates were used as potential determinants of belonging to a given trajectory: age, gender, body mass index (BMI), smoking status (never, former and current smoker), comorbidities (diabetes, asthma, cardiovascular diseases, and hypertension), regular treatments at time of study inclusion, antibiotics taken in the 2 months before COVID- 19 infection and disease severity at inclusion proxied by the total number of symptoms.

We fitted an univariate logistic regression model on each imputed dataset and pooled the models for a single set of estimates following the Rubin’s rules to explore the association of a characteristic and the different trajectories. Each characteristic was explored with the adjustment of the other characteristics in the model. Regression coefficients (Beta) with 95% Confidence intervals were estimated.

### Missing values

We did not need to impute missing values for the trajectories modeling as we only included participants who responded to the entire dataset of 10 symptoms. However, participants were included in this study if they completed at least 2 out of the 4 timepoints.

We imputed the missing values in the covariates and generated 45 imputed datasets.

We performed all the analysis with the R version 4.3.0 [23]. We used lcmm R package for trajectory analysis, the mice R package for missing covariate values imputation, and the ggplot2 R package.

### Sensitivity analysis

#### Impact of missing timepoints on total score trajectories

To assess the impact of missing timepoints on the total score trajectories, we compared the trajectories obtained on data from the 555 participants who completed at least baseline data and one monthly questionnaire with trajectories obtained on 84 participants who completed the 4 timepoints.

#### Quality of life evaluation

We described the impact of symptoms on quality of life in a subpopulation of 141 participants who completed the M24 questionnaire.

Sleep quality was assessed using the PSQI questionnaire. A categorical variable was generated using the PSQI score. Poor sleep was defined as PSQI total score ≥ 5 [24].

The respiratory quality of life was assessed using the VQ11 questionnaire, initially developed for COPD patients. One global score and 3 sub-scores (functional, psychological and relational) were calculated as described elsewhere and categorical variables were generated [25, 26]. An altered respiratory quality of life was defined as VQ11 global score ≥ 22, an altered physical autonomy as functional component ≥ 8, an altered psychological quality of life as psychological component ≥ 10 and an altered relational quality of life as relational component ≥ 10.

The stress level was assessed using the Perceived Stress Scale 4 (PSS 4) questionnaire. The final score ranged from 0 to 16, the highest score corresponding to a higher stress level. A PSS4 score of 6 and above was used to identify participants with high levels of stress [27].

The Fatigue Severity Scale (FSS9) which has recently been validated in COVID- 19 population was used to measure the fatigue level [28]. The FSS9 score corresponded to the mean of the scores from the 9 items. A high level of fatigue was defined as a total score ≥ 36.

The Generalized Anxiety Disorder 7-item (GAD7) has been used to grade the level of anxiety. A score above or equal to a cut-off of 10 was considered to identify generalized anxiety disorder [29].

### Descriptive statistics

We described the continuous variables, when the skewness was between − 1 and 1, as mean ± SD, otherwise, as median [min,max], while the categorical variables as numbers (percentage). To determine the differences of distribution we used the student t-test for normally distributed continuous variables, the Wilcoxon test for non normally distributed continuous variables and the Fisher’s exact test for categorical variables.

### Trajectories modeling

We used latent class mixed modeling (LCMM) [30] to identify and describe distinct trajectories in the evolution of the total symptom score and of individual symptoms from baseline to M24. This method characterizes trajectories in repeated measurements, with the assumption that several underlying subpopulations or latent classes exist. The LCMM does not require the same number of measurements per participant or measurement time points. The time metric used was the time in days from baseline. We first tested different link functions, including linear and splines with different number of nodes and nodes location, to identify the best-fitting model with one class, which had the lowest Bayesian information criterion (BIC). We then estimate the model with selected link function with two to four classes to determine the optimal number of latent trajectories, appraising the entropy of the model. We applied a gridsearch to ensure the convergence of the model. We did not include covariates to predict latent class membership.

## Results

### Study population characteristics

The study population was composed of 51.5% of women, mean age was 41.6 years (± 12.6), and mean BMI was 25.1 kg/m^2^ [16.7,55.1]. Thirty-two percent of the participants took at least one regular treatment and 6.3% had at least 2 comorbidities prior COVID- 19 infection. The majority of study participants were not hospitalized during acute infection (545/555; 98%).

The most frequent treatments were anti-hypertensive (10.4%), antibiotics (10.4%), and anti-cholesterol (7.4%).

### Total symptom score trajectories

Based on the lowest BIC and the highest entropy, the optimal number of total score trajectories was identified as 2 (see Supplementary Table 1, additional file 2).

**Fig. 1:**
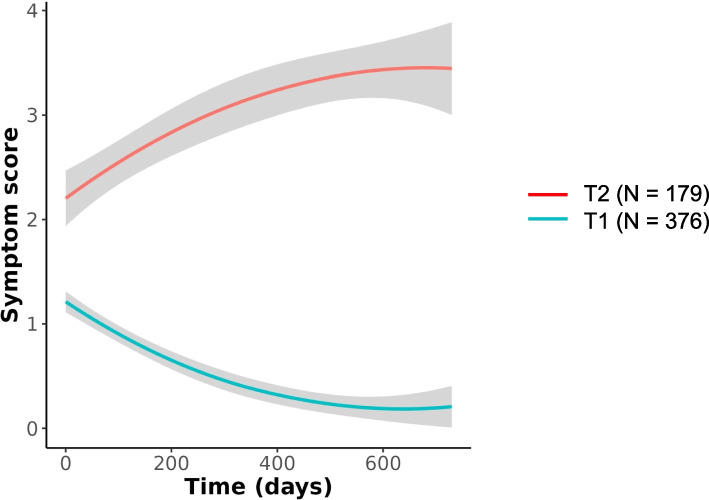
Total symptom score trajectories. Total symptom score evolution in T1 “Mild symptoms, fast resolution”, and T2 “Elevated and persisting symptoms”, from baseline up to 24 months after (in days). The grey areas show the 95% confidence intervals

The total score trajectories were named according to their characteristics: T1, mild symptoms, fast resolution, and T2, elevated and persisting symptoms. The trajectories are presented in Fig. 1.

The number of participants in each trajectory was 376/555 (67.7%) in T1 and 179/555 (32.3%) in T2. Participants in the T2 “Elevated and persisting symptom” trajectory were more frequently female (61.5% vs 46.8%), had a higher BMI (26.3 vs 24.7), were older (44 vs 40.5 years), had more frequently more than 2 comorbidities (10.6% vs 4.3%), and took more frequently at least 1 chronic medication (44.7% vs 26.3%) than participants in the T1 “Mild symptoms, fast resolution” trajectory.

Participants characteristics in total study population and in each trajectory are summarized in Table 1.

**Table 1:** Participant's individual characteristics

Variable Mean (±SD) for normally distributed continuous variables; Median [min;max] for non-normally distributed continuous variables; N (%) for categorical variables	Total study population *N*= 555	T1: Mild symptoms. rapid resolution *N* = 376 (67.7%)	T2: Elevated and persisting symptoms *N*= 179 (32.3%)	*P*-Value
BMI (kg/m²)	25.1[16.7;55.1]	24.7[16.7;55.1]	26.3[18.4;48.9]	< 0.001
Female (yes)	286(51.53)	176(46.81)	110(61.45)	0.001
Age (years)	41.6 ± 12.6	40.5 ± 12.4	44 ± 12.7	0.002
At least 2 comorbidities (yes)	35(6.31)	16(4.26)	19(10.61)	0.008
Current smoker (yes)	90(16.25)	56(14.89)	34(19.10)	0.275
Total symptom score initial infection	1[1;8]	1[0;6]	2[0;8]	< 0.001
Total symptom score M12	0[0;9]	0[0;4]	3[0;9]	< 0.001
Total symptom score M15	1[0;10]	0[0;4]	3[1;10]	< 0.001
Total symptom score M24	1[0;9]	0[0;2]	3[1;9]	< 0.001
Participants with at least 1 medication before COVID- 19 infection	179 (32.3)	99 (26.3)	80 (44.7)	< 0.001
Sleep aids	6(1.08)	0(0.00)	6(3.35)	0.001
Anti hypertensive	58(10.45)	28(7.45)	30(16.76)	0.002
Anti pain/inflammation	14(2.53)	4(1.07)	10(5.59)	0.003
Anti cholesterol	41(7.40)	21(5.60)	20(11.17)	0.024
Diabetes treatment	15(2.70)	6(1.60)	9(5.03)	0.026
Treatment for anxiety	23(4.14)	11(2.93)	12(6.70)	0.042
Antibiotic in the 2 months before COVID- 19 initial infection	57(10.36)	33(8.85)	24(13.56)	0.100
Anti coagulant	18(3.25)	9(2.40)	9(5.03)	0.125
Anti depressant	22(3.96)	12(3.19)	10(5.59)	0.243
Anti convulsant	6(1.08)	3(0.80)	3(1.68)	0.393

*P*-values between T2 and T1 were calculated using the student t-test for normally distributed continuous variables, the Wilcoxon test for non normally distributed continuous variables and the Fisher's exact test for categorical variables

**Fig. 2:**
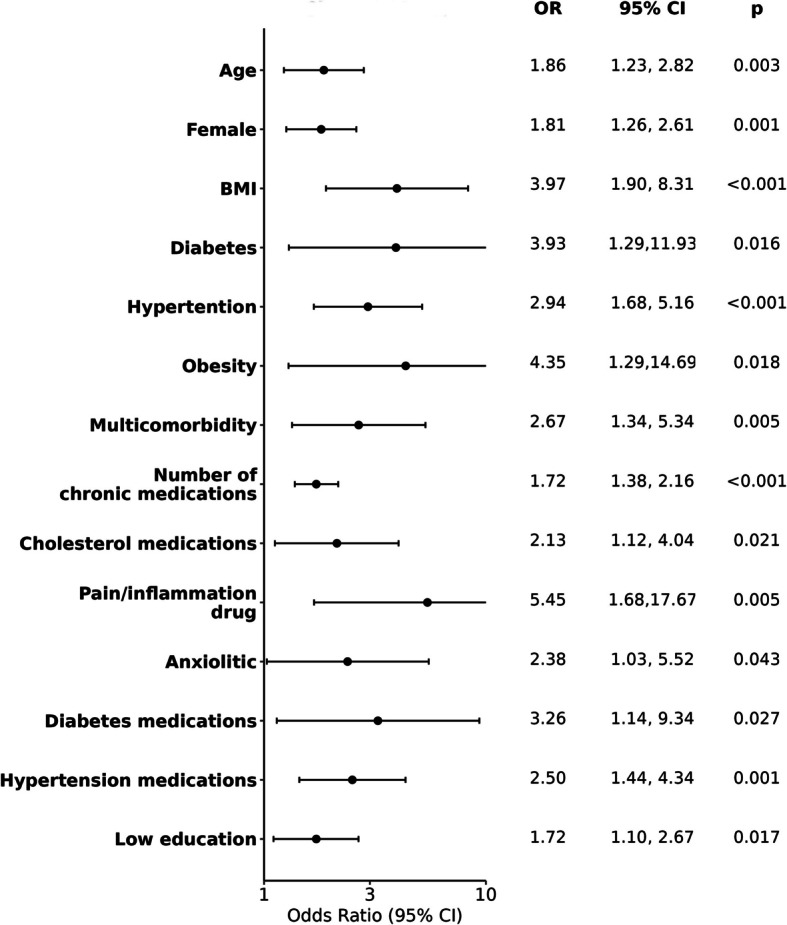
Determinants of being in T2 (Elevated and persisting symptoms) vs T1 (Mild symptoms, fast resolution)

The main determinants of experiencing a T2 “Elevated and persisting symptoms” trajectory were older age, being a female, higher BMI, multi comorbidities, diabetes, hypertension, the number and type of chronic medications (for pain, diabetes in particular) (see Fig. 2).

**Fig. 3:**
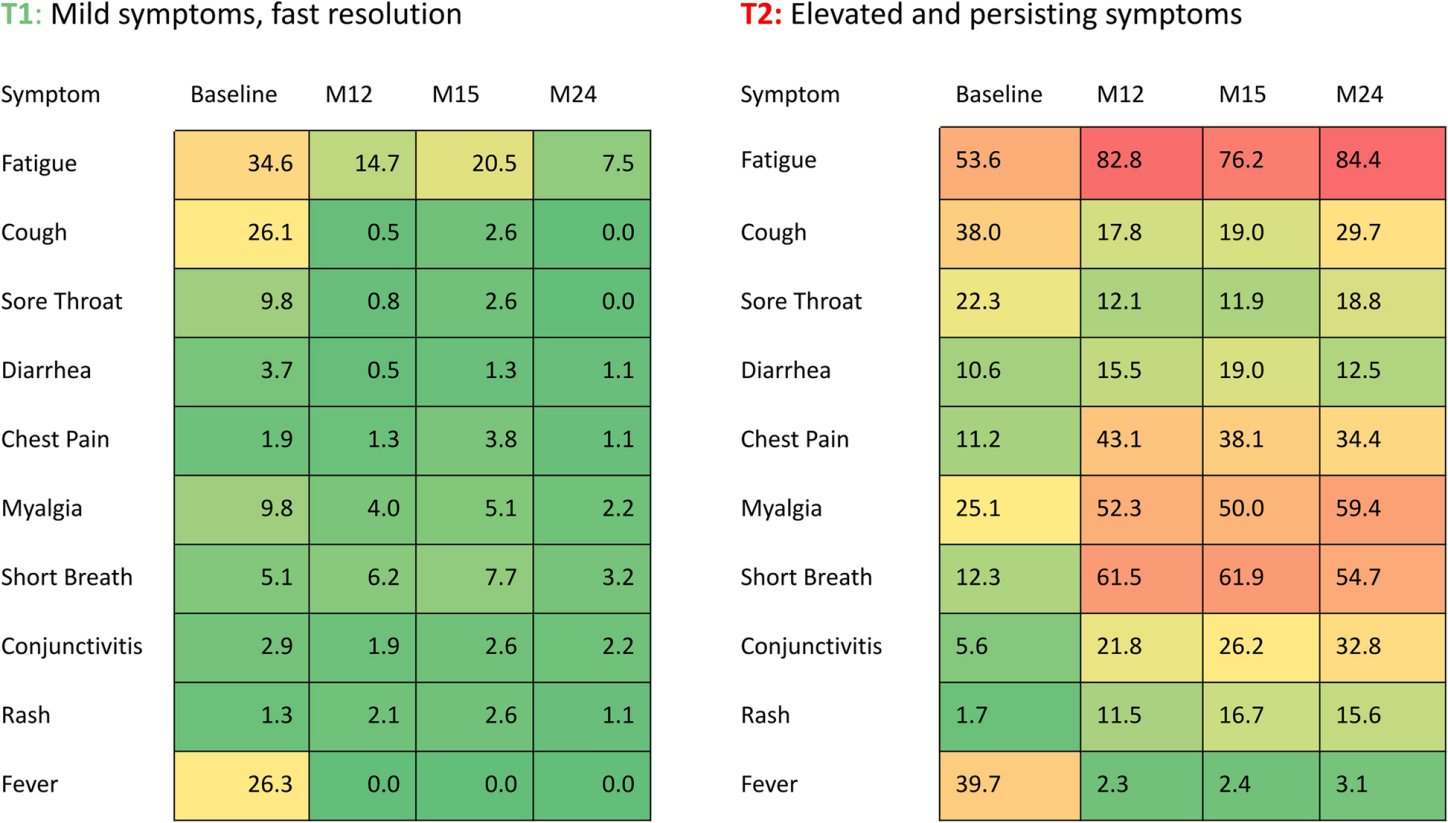
Symptom frequencies in T1 and T2 trajectories. Symptom frequencies are provided for each trajectory at baseline, M12, M15, and M24 (%)

When exploring symptom frequencies at each time point in the 2 trajectories we observed that fatigue, cough and fever were the most frequent symptoms at baseline in both trajectories. Symptom frequencies decreased in T1 from baseline until M24, at various speeds. In particular, fatigue decreased more slowly than couch or fever. In T2, fatigue, pain-related symptoms (chest pain, myalgia), shortness of breath, and conjunctivitis frequencies increased between baseline and M12 and remained elevated until M24. Cough frequency decreased between baseline and M12, and increased again between M15 and M24. Symptom frequencies in both trajectories are shown in Fig. 3.

### Individual symptom trajectories

**Fig. 4:**
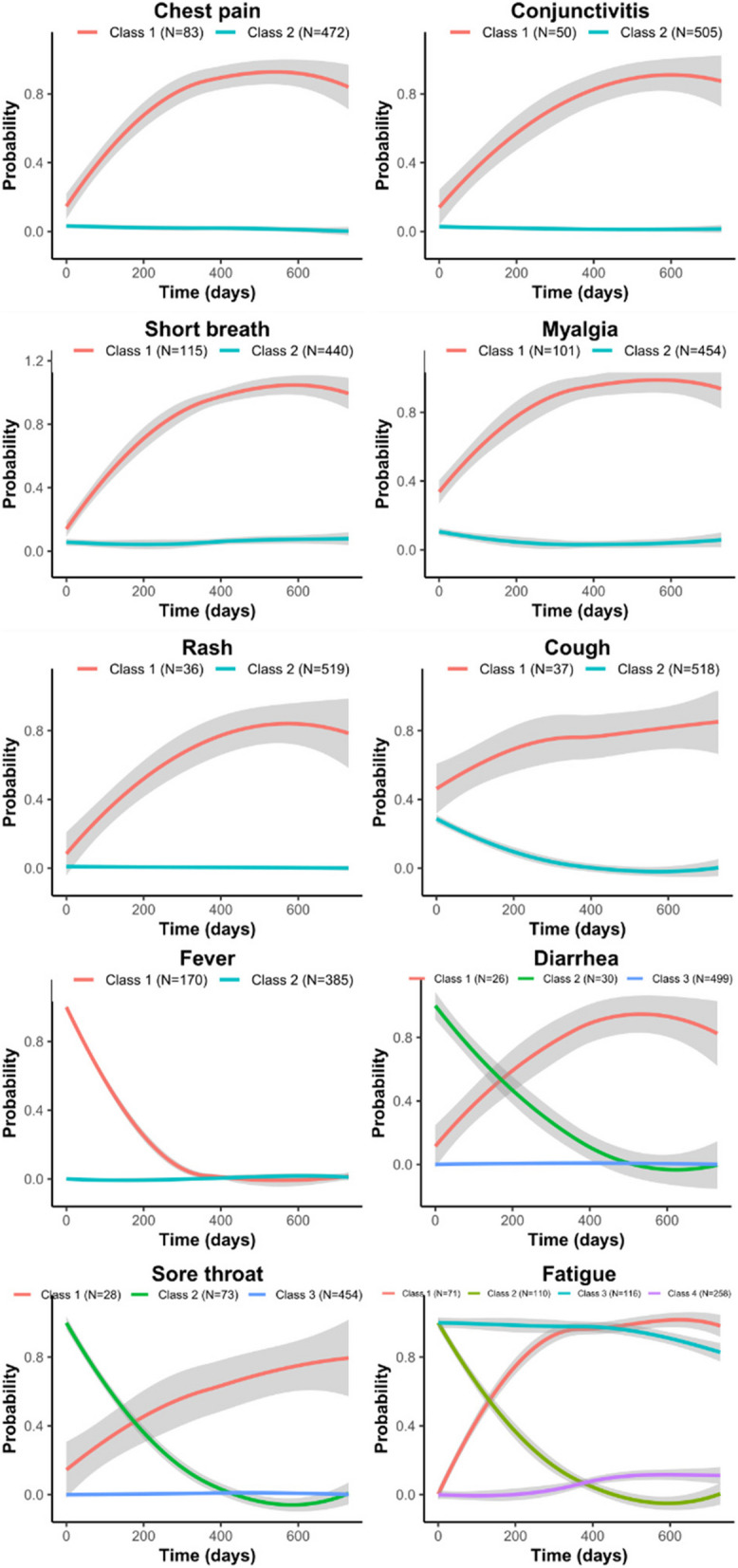
Individual symptoms trajectories. Individual symptom trajectories were modeled for the 555 participants from baseline until month 24 (in days). For each symptom the optimal number of classes was defined using the model with the lowest BIC and the highest entropy. Different numbers of classes (or trajectories) were obtained depending on the symptom and were named class 1, class 2, etc

Individual symptom trajectories from baseline up to M24 were also identified and are summarized in Fig. 4. For each symptom, the optimal number of total score trajectories was determined based on the lowest BIC and the highest entropy. Briefly, some symptoms evolved following 2 trajectories, one trajectory remaining at a low level and the other one increasing over time (chest pain, conjunctivitis, shortness of breath, myalgia, rash and cough). Diarrhea and sore throat evolved following 3 trajectories, one being low, one increasing and one decreasing. Fever and fatigue had particular patterns of evolution. Fever followed 2 trajectories, one including participants with low level and the other one with fever decreasing in a fast way after baseline.

Fatigue was the most complex symptom in terms of individual trajectories as we identified 4 different trajectories: one with half of the participants experiencing low level of fatigue, but with a slight increase over time, the second trajectory with initial low level of fatigue but increasing and remaining at a high level until M24, the third one with initial high level of fatigue but decreasing rapidly over time, and the last one with fatigue being highly present from baseline until M24. Individual characteristics of participants in the 4 fatigue trajectories are provided in supplementary Table 2 (see additional file 3).

### Sensitivity analysis

The trajectories obtained on 84 participants with complete data at each timepoint were similar to those obtained on the population of 555 participants described above (See supplementary Fig. 1, additional file 4).

We also described the quality of life of 138 participants who completed the month 24 questionnaire, in the total population and in the 2 trajectories. In brief, participants in the T2 “Elevated and persisting symptoms” trajectory had higher stress, fatigue and anxiety levels, and were more likely to experience poor sleep quality and poor respiratory quality of life than participants in the T1 “Mild symptoms, fast resolution” trajectory. They also less frequently recovered a similar life rhythm and professional activity as before SARS-CoV- 2 infection, and they were more likely to experience a worsening of their relationships with their family or friends (see Table 2).

**Table 2:** Quality of life 24 months after initial COVID- 19 infection, in a subpopulation of 141 participants who completed the M24 questionnaire

Variable	Total study population (*N*= 141)	T1: Mild symptoms, fast resolution (*N* = 87)	T2: Elevated and persisting symptoms (*N*= 54)	*p* value (T1 vs T2)
Stress level (PSS4 score)	5.3 ± 3.4	4.5 ± 3.2	6.6 ± 3.4	0.001
Participants with high stress level N(%)	62(43.97)	31(35.63)	31(57.41)	0.015
Fatigue level (FSS9 score)	29.1 ± 16.4	22.1 ± 13.3	40.3 ± 14.8	< 0.001
Participants with high fatigue level, N(%)	52(36.88)	17(19.54)	35(64.81)	< 0.001
Anxiety level (GAD7 score)	4[0, 21]	1[0,21]	6.5[0,21]	< 0.001
Participants with high anxiety level, N(%)	17(12.06)	4(4.60)	13(24.07)	0.001
Sleep (PSQI)	5[0,20]	5[0,14]	8[2,20]	< 0.001
Poor sleep, N(%)	86(60.56)	45(51.14)	41(75.93)	0.004
Respiratory quality of life (VQ11)	14[11,47]	12[11,34]	19.5[11,47]	< 0.001
Altered respiratory quality of life, N(%)	27(19.15)	4(4.60)	23(42.59)	< 0.001
Life rhythm recovered as before COVID- 19 (Yes)	132(88.00)	88(97.78)	44(73.33)	< 0.001
Professional activity unrecovered, N(%)	6(4.00)	1(1.11)	5(8.33)	0.038
Relationship with family or friends worsened, N(%)	10(6.67)	1(1.11)	9(15.00)	0.001
No symptoms anymore, N(%)	75(50.00)	69(76.67)	6(10.00)	< 0.001
Symptoms occurring under the form of crisis, N(%)	40(26.67)	10(11.11)	30(50.00)	< 0.001
Symptoms being constants, N(%)	35(23.33)	11(12.22)	24(40.00)	< 0.001

*P*-values are determined using the student t-test or Wilcoxon test for continuous variables

**Fig. 5:**
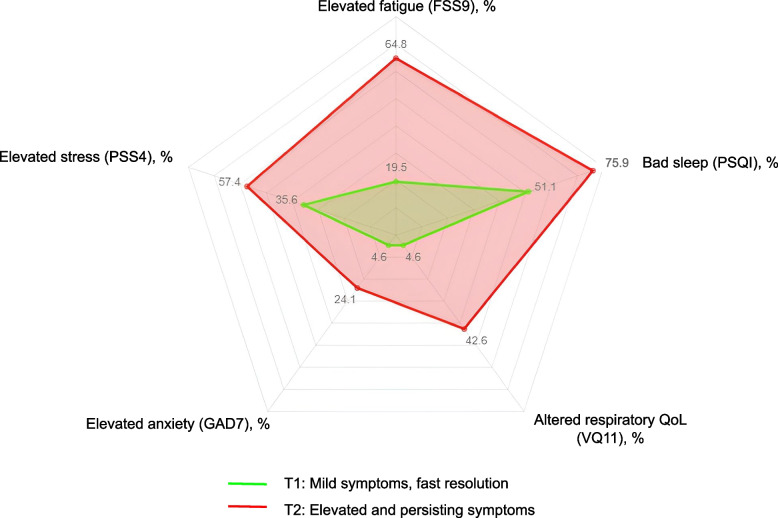
Participants with altered quality of life at M24. Radar diagram showing the percentage of participants with high levels of fatigue, stress, anxiety and with poor sleep and respiratory quality of life in each trajectory using the specific cut-off score of each scale

The percentage of participants above the cut-off in each of the PSS4, FSS9, GAD7, PSQI and VQ11 scales is summarized in Fig. 5 and shows a degradation of these 5 indicators in participants from the T2 “Elevated and persisting symptoms” trajectory.

The viral load was measured in nasopharyngeal swabs from 172 participants, collected during the study inclusion visit taking place within 5 days after the initial confirmation of infection. Among them, 145 (84.3%) still had detectable levels of viral RNA, and 129 (75%) had a measurable viral load. Viral RNA levels were below LoQ cut-off for 16 participants preventing viral load calculation.

The median viral load at baseline was 1.2E6 [1.4E3,1.8E9] RNA copies/ml in the entire cohort, and was higher in T2 than in T1 (2.6E6 [1.5E3,1.8E9] and 9.3E5[1.4E3,1.3E9] RNA copies/ml respectively; *p* = 0.139).

## Discussion

In this study we described the evolution of a score based on 10 COVID- 19-related symptoms, from the initial infection up to 24 months after. We have observed two trajectories, with one third of our study participants experiencing a T2 “Elevated and persisting symptoms” trajectory, with some symptoms having increasing frequencies until month 24, and having their quality of life heavily impacted. Fatigue was the most frequent symptom in both total score trajectories and we identified 4 trajectories of fatigue taken individually.

### Comparison with literature

Although an increasing number of studies describe Long Covid prevalence, subphenotypes and related symptoms at 12 or 24 months [17, 31–33], few of them aimed at modeling the long-term trajectories of Long COVID evolution [19, 21]. Our results are in coherence with these studies which showed also that a subpopulation of people with Long COVID experienced very long lasting symptoms with little recovery over time. Other studies focused on trajectories from specific symptoms like neurological or respiratory symptoms [34–36].

We found that fatigue was predominant in both trajectories. Its frequency increased over time in the T2 “Elevated and persisting symptoms”, whereas in the T1 “Mild symptoms, fast resolution” trajectory it remained on a higher level than other symptoms until M15 and decreased at M24. Looking at fatigue independently from other symptoms we identified 4 different trajectories, with 34% of our participants with either a high and persisting level of fatigue from the acute infection until 24 months after, or an initial low level of fatigue importantly increasing until month 12 and reaching a maximum between month12 and month 24. This tendency of fatigue persistence has been recently described in a recent meta-analysis on the neurological symptoms of Long COVID at 12 months [35] and another study also described a worsening of fatigue over time [37].

Being a woman and of higher age were risk factors to experience the T2 persisting Long COVID trajectory. We also showed that preexisting comorbidities like diabetes, obesity and hypertension, and associated treatments, but also treatments for pain, inflammation and anxiolytics, were associated with a higher risk of developing a severe form of Long COVID. These findings are in line with results previously described [21, 38].

There are few studies describing the quality of life of people with Long COVID, and they generally focus on overall quality of life using questionnaires like SF12, EQ- 5D- 3L, or EQ- 5D- 5L [39, 40] or on only one specific aspect like fatigue [41]. A recent study described the quality of life of people with Long COVID at a median time of 197.5 days after initial infection using various scales (including GAD7, PHQ9, MOCA) and showed subpopulations with a higher impact on quality of life [17]. Our study is providing additional information on the multiple aspects of quality of life that are impacted by Long COVID 24 months after acute infection. We showed that being in the T2 “Elevated and persisting symptoms” was associated with a multidimensional alteration of quality of life (altered sleep and respiratory quality of life, increase of fatigue, stress and anxiety).

The impaired respiratory quality of life observed at month 24 in people belonging to the T2 highly persisting trajectory could be explained by a limited recovery in lung function 2 years after initial infection [34].

Participants in the T2 persisting trajectory had a higher SARS-CoV- 2 viral load during acute infection, even though this result was not statistically significant due to the low number of data available. Previously, some studies found no relation between viral load and early COVID- 19 clinical outcomes [42, 43], however another study suggested a correlation between higher viral load during acute infection and Long COVID [44]. It would be of interest to deeper investigate this finding as it may provide new insight on Long COVID determinants and biological mechanisms.

### Strengths and limitations

Our study has several strengths. First, all study participants had an initial PCR-confirmed SARS-CoV- 2 infection and were prospectively followed up to 24 months after. Trajectories have been modeled based on 10 symptoms collected systematically at each timepoint from day 0 to month 24. Finally, study participants were in majority non hospitalized individuals, enhancing the result’s generalizability since the majority of people with Long COVID undergo mild infections.

This study also has some limitations. The high number of participants who did not complete the questionnaire at months 15 and 24 might have led to an overestimation of Long COVID symptoms at 24 months, as people who completed the questionnaire were experiencing more symptoms than participants who completed only the questionnaire at 12 months. However, our sensitivity analysis on participants who completed the full set of questionnaires showed similar trajectories, confirming the reliability of our results.

Symptoms were self-reported, and we could not fully assert that reported symptoms were linked to Long COVID and we could not exclude that other concomitant health issues could have interfered. All the participants in this study were infected with pre-Omicron variants, thus our results may not be generalized to people infected by the Omicron variant. Finally, information on vaccination status was not available and may be of interest to explain the different trajectories.

## Conclusions

Our findings demonstrated a high diversity in the long-term evolution of Long COVID. One-third of study participants are still suffering from symptoms 24 months after the acute illness with a significant impact on various dimensions of their quality of life. This work underlined the need to identify the individuals most vulnerable to long-term sequelae to develop tailored care interventions.

## Supplementary Information

Additional file 1

Additional file 2: Supplementary Table 1: Determination of the optimal class number. The optimal number of classes is determined by the lowest BIC and the highest entropy

Additional file 3: Supplementary Table 2: Participant's individual characteristics in fatigue trajectories

Additional file 4: Supplementary Fig. 1: Complete case analysis: total symptom score evolution in T1 and T2 from baseline up to 24 months afterfor 84 participants who completed the 4 timepoints

## Data Availability

The datasets used and/or analyzed during the current study are available from the corresponding author on reasonable request.
