# Supplementary material for: Trajectories of persisting Covid- 19 symptoms up to 24 months after acute infection: findings from the Predi-Covid cohort study
Source: BMC Infect Dis. 2025 Apr 25;25:603. doi: 10.1186/s12879-025-11023-0 (PMC12023393; doi:10.1186/s12879-025-11023-0)
Supplement: Supplementary file 1 — Additional file 1 [file 12879_2025_11023_MOESM1_ESM.pdf]

Predicovid (adult participants)- Questionnaire M12-15-24

Have you noticed the following symptoms or illnesses since your Covid-19 diagnosis?

Responses :

- yes and I still feel it today
- yes, I had it but I no longer have it o conditional question: “This symptom disappeared _ _ weeks after diagnosis
- no, I have never had this symptom

ENT symptoms :

1) loss of taste
2) loss of smell
3) Runny nose, cold or rhinitis
4) Pain in the sinuses
5) Pain in the ears
6) Sore throat, feeling of constriction in the throat or pain when you swallow
7) Voice alteration

Neurological and ocular symptoms:

8) Tremor of the hands or limbs
9) Headache
10) Migraines
11) Mental confusion (slowing down of thought and / or reasoning)
12) Feeling sick or dizzy (dizziness, fainting)
13) Epileptic attacks / convulsions
14) Balance disorders
15) Memory loss / short-term memory impairment
16) Tiredness in your eyes
17) Hallucinations
18) Sensitivity to light (photophobia)
19) Conjunctival inflammation, itchy eyes or red / flushed eyes
20) Speech disorders

General symptoms:

21) Fatigue
22) Irritability, exacerbated nervousness
23) Anxiety
24) Depression
25) Sweats / chills
26) Fever (> = 38 ° C)
27) loss of appetite
28) Unintentional weight loss (over 3 kg)
29) recurrent feeling of thirst
30) Tingling / pins and needles / numbness sensations in the limbs or on the skin
31) Muscle or joint pain in the upper limbs
32) Muscle or joint pain in the lower limbs
33) back pain
34) Newly appeared allergy (if yes: what type of allergy / free field or list)

35) hair loss
36) Difficulty walking

Cardio-respiratory symptoms or diseases:

37) Shortness of breath / shortness of breath
38) Feeling of tightness in the chest
39) Dry cough
40) Fatty cough
41) Tachycardia (abnormally high heart rate while resting or during low-intensity activities)
42) Arrhythmia / Palpitations (irregular heartbeat or feeling that the heart is beating very hard)
43) Myocarditis
44) Pericarditis
45) Heart failure
46) Myocardial infarction
47) Burning sensation in the chest
48) Chest pain
49) Wheezing
50) Bloody sputum
51) Thrombosis

Gastrointestinal symptoms:

52) Nausea
53) Vomiting
54) Diarrhea (occurrence of watery stools)
55) Constipation
56) Stomach burn
57) Other abdominal pain

Vascular / lymph node symptoms or diseases:

58) Hypertension
59) Hypotension
60) Stroke
    - If yes: Ischemic or hemorrhagic?
61) Lymphadenopathy (swollen, painful or inflamed lymph nodes)
62) Circulation disorders (swollen veins, heavy legs, etc.)
63) Spontaneous hematomas

Urinary symptoms:

64) Urinary pain
65) Recurrent urinary tract infections
66) Need for dialysis

Skin symptoms:

67) Skin rashes / lesions
68) Dry skin
69) Blue / purple / white or swollen fingers or toes

70) Other symptoms:

General questions

71) How do you feel today? (I feel good / I feel tired) / I feel bad)

72) Taking into account all the symptoms that can be attributed to Covid-19 that you have experienced in the last 30 days (frequency, intensity, impact on your life), would you say that you could live long term in your current state of health? (Yes/No)

73) Do the symptoms you have mentioned occur during crisis, with phases of improvement or worsening?
- ○ Yes, with daily crises
- ○ Yes, with at least 1 crisis per week
- ○ Yes, but less than one crisis per week
- ○ No, my symptoms are constant over time
- ○ I have no symptoms

74) How long do these crisis usually last?
- ○ Less than a day
- ○ More than a day but less than a week
- ○ More than a week but less than a month
- ○ I don't have crisis

75) What is your current pain level? (Rate from 1 to 10)

76) Since filling out your last questionnaire, have you consulted for a reason related to Covid19? Yes No ->

If yes, for what reason?

77) Have you taken a lifestyle comparable to the one you had before the onset of symptoms associated with Covid19? Yes/No

If no, why?

78) Did you return to your normal occupation? Yes / No / I am retired or unemployed

If no, have you been on sick leave following complications from Covid19?

79) Since the diagnosis of Covid19 or the onset of symptoms associated with Covid19, have your relationships with those around you (family, friends): Worsened / Stayed the same / Improved

if worsened or improved: Why?

80) Since the diagnosis of Covid19 or the onset of symptoms associated with Covid19, would you say that your : appetite/physical activity/sleep: Worsened / Stayed the same / Improved

81) Do you currently benefit or have you benefited from a consultation dedicated to the Long Covid syndrom? Yes/no/Don’t know

- If yes: have you been offered rehabilitation? Yes/no/Don't know
- If yes: at which institution? Rehazenter/Mondorf/CHNP/Other, precise

82) Since the diagnosis of Covid19 or the onset of symptoms associated with Covid19, have you noticed any changes in your menstrual cycles? Yes/No/Don't know/ Not applicable

- If yes: heavier periods / irregular periods / early menopause/ Don't know

83) **Sleep, PSQI scale**

1. During the past month, when have you usually gone to bed at night?

   USUAL BED TIME ______________________

2. During the past month, how long (in minutes) has it usually take you to fall asleep each night?

   NUMBER OF MINUTES ______________________

3. During the past month, when have you usually gotten up in the morning?

   USUAL GETTING UP TIME ______________________

4. During the past month, how many hours of actual sleep did you get at night? (This may be different than the number of hours you spend in bed.)

   HOURS OF SLEEP PER NIGHT ______________________

5. During the past month, how often have you had trouble sleeping because you...

	Not during the past month	Less than once a week	Once or twice a week	Three or more times a week
(a) ...cannot get to sleep within 30 minutes	☐	☐	☐	☐
(b) ...wake up in the middle of the night or early morning	☐	☐	☐	☐
(c) ...have to get up to use the bathroom	☐	☐	☐	☐
(d ...cannot breathe comfortably	☐	☐	☐	☐
(e) ...cough or snore loudly	☐	☐	☐	☐
(f) ...feel too cold	☐	☐	☐	☐
(g) ...feel too hot	☐	☐	☐	☐
(h) ...had bad dreams	☐	☐	☐	☐
(i) ...have pain	☐	☐	☐	☐

(j) Other reason(s), please describe

____

____

How often during the past month have you had trouble sleeping because of this?	☐	☐	☐	☐

	Very good	Fairly good	Fairly bad	very bad
6. During the past month, how would you rate your sleep quality overall?	☐	☐	☐	☐

	Not during the past month	Less than once a week	Once or twice a week	Three or more times a week
7. During the past month, how often have you taken medicine (prescribed or "over the counter") to help you sleep?	☐	☐	☐	☐
8. During the past month, how often have you had trouble staying awake while driving, eating meals, or engaging in social activity?	☐	☐	☐	☐

	No problem at all	Only a very slight problem	Somewhat of a problem	A very big problem
9. During the past month, how much of a problem has it been for you to keep up enough enthusiasm to get things done?	☐	☐	☐	☐

	No bed partner or roommate	Partner/ roommate in other room	Partner in same room, but not same bed	Partner in same bed
10. During the past month, how much of a problem has it been for you to keep up enough enthusiasm to get things done?	☐	☐	☐	☐

If you have a roommate or bed partner, ask him/her how often in the past month you have had...

	Not during the past month	Less than once a week	Once or twice a week	Three or more times a week
(a) ...loud snoring	☐	☐	☐	☐
(b) ...long pauses between breaths while asleep	☐	☐	☐	☐
(c) ...legs twitching or jerking while you sleep	☐	☐	☐	☐
(d) ...episodes of disorientation or confusion during sleep	☐	☐	☐	☐
(e) Other restlessness while you sleep; please describe	☐	☐	☐	☐

84) Quality of life (SF12)

1. In general, would you say your health is:

☐1 Excellent ☐2 Very good ☐3 Good ☐4 Fair ☐5 Poor

The following questions are about activities you might do during a typical day. Does your health now limit you in these activities? If so, how much?

	**YES, limited a lot**	**YES, limited a little**	**NO, not limited at all**
2. **Moderate activities** such as moving a table, pushing a vacuum cleaner, bowling, or playing golf.	☐1	☐2	☐3
3. Climbing **several** flights of stairs.	☐1	☐2	☐3

During the past 4 weeks, have you had any of the following problems with your work or other regular daily activities as a result of your physical health?

	**YES**	**NO**
4. **Accomplished less** than you would like.	☐1	☐2
5. Were limited in the **kind** of work or other activities.	☐1	☐2

During the past 4 weeks, have you had any of the following problems with your work or other regular daily activities as a result of any emotional problems (such as feeling depressed or anxious)?

	**YES**	**NO**
6. **Accomplished less** than you would like.	☐1	☐2
7. Did work or activities **less carefully than usual**.	☐1	☐2

8. During the past 4 weeks, how much did pain interfere with your normal work (including work outside the home and housework)?

☐1 Not at all ☐2 A little bit ☐3 Moderately ☐4 Quite a bit ☐5 Extremely

These questions are about how you have been feeling during the past 4 weeks.
For each question, please give the one answer that comes closest to the way you have been feeling.

How much of the time during the past 4 weeks…

	All of the time	Most of the time	A good bit of the time	Some of the time	A little of the time	None of the time
9. Have you felt calm & peaceful?	☐1	☐2	☐3	☐4	☐5	☐6
10. Did you have a lot of energy?	☐1	☐2	☐3	☐4	☐5	☐6
11. Have you felt down-hearted and blue?	☐1	☐2	☐3	☐4	☐5	☐6

12. During the past 4 weeks, how much of the time has your physical health or emotional problems interfered with your social activities (like visiting friends, relatives, etc.)?

☐1 All of the time ☐2 Most of the time ☐3 Some of the time ☐4 A little of the time ☐5 None of the time

85) Respiratory quality of life (VQ11)

I suffer from breathlessness

I am worried about my respiratory condition

I feel my entourage (family, friends, etc.) misunderstands me

My respiratory condition prevents me from moving about as easily as I would like

I feel sleepy during the day

I feel unable to achieve my objectives

I quickly get tired when doing day-to-day activities

Physically, I am dissatisfied with what I can do

My respiratory disease disrupts my social life

I feel sad

My respiratory condition restricts my emotional life

86) Generalized Anxiety Disorder (GAD7)

1. Over the last two weeks how often have you been bothered by any of the following problems?

	**Not at all** (0)	**Several days** (1)	**More than half the days** (2)	**Nearly every day** (3)
a. Feeling nervous, anxious or on edge	☐	☐	☐	☐
b. Not being able to stop or control worrying	☐	☐	☐	☐
c. Worrying too much about different things	☐	☐	☐	☐
d. Trouble relaxing	☐	☐	☐	☐
e. Being so restless that is hard to sit still.	☐	☐	☐	☐
f. Becoming easily annoyed or irritable	☐	☐	☐	☐
g. Feeling afraid as if something awful might happen	☐	☐	☐	☐

Total Score: __________

87) Perceived stress scale (PSS4)

The questions in this scale ask you about your feelings and thoughts during THE LAST MONTH. In each case, please indicate your response by placing an "X" over the square representing HOW OFTEN you felt or thought a certain way.

	Never 0	Almost Never 1	Sometimes 2	Fairly Often 3	Very Often 4
1. In the last month, how often have you felt that you were unable to control the important things in your life?	☐	☐	☐	☐	☐
2. In the last month, how often have you felt confident about your ability to handle your personal problems?	☐	☐	☐	☐	☐
3. In the last month, how often have you felt that things were going your way?	☐	☐	☐	☐	☐
4. In the last month, how often have you felt difficulties were piling up so high that you could not overcome them?	☐	☐	☐	☐	☐

88) Fatigue severity scale (FSS9)

**During the past week, I have found that:**	**Disagree ⟵**					**⟶ Agree**	
1. My motivation is lower when I am fatigued.	1	2	3	4	5	6	7
2. Exercise brings on my fatigue.	1	2	3	4	5	6	7
3. I am easily fatigued.	1	2	3	4	5	6	7
4. Fatigue interferes with my physical functioning.	1	2	3	4	5	6	7
5. Fatigue causes frequent problems for me.	1	2	3	4	5	6	7
6. My fatigue prevents sustained physical functioning.	1	2	3	4	5	6	7
7. Fatigue interferes with carrying out certain duties and responsibilities.	1	2	3	4	5	6	7
8. Fatigue is among my three most disabling symptoms.	1	2	3	4	5	6	7
9. Fatigue interferes with my work, family, or social life.	1	2	3	4	5	6	7
