# Supplementary material for: Trajectories of persisting Covid- 19 symptoms up to 24 months after acute infection: findings from the Predi-Covid cohort study
Source: BMC Infect Dis. 2025 Apr 25;25:603. doi: 10.1186/s12879-025-11023-0 (PMC12023393; doi:10.1186/s12879-025-11023-0)
Supplement: Supplementary file 2 — Additional file 2: Supplementary Table 1: Determination of the optimal class number. The optimal number of classes is determined by the lowest BIC and the highest entropy [file 12879_2025_11023_MOESM2_ESM.pdf]

Supplementary table 1: Determination of the optimal class number

	BIC	conv	loglik	Entropy
nclass_2	3877.439	1	-1900.806	0.6610324
nclass_3	3896.396	1	-1900.806	0.3558408
nclass_4	3915.353	1	-1900.806	0.2913690

The optimal number of classes is determined by the lowest BIC and the highest entropy.
