# Supplementary material for: Trajectories of persisting Covid- 19 symptoms up to 24 months after acute infection: findings from the Predi-Covid cohort study
Source: BMC Infect Dis. 2025 Apr 25;25:603. doi: 10.1186/s12879-025-11023-0 (PMC12023393; doi:10.1186/s12879-025-11023-0)
Supplement: Supplementary file 4 — Additional file 4: Supplementary Fig. 1: Complete case analysis: total symptom score evolution in T1 and T2 from baseline up to 24 months afterfor 84 participants who completed the 4 timepoints [file 12879_2025_11023_MOESM4_ESM.pdf]

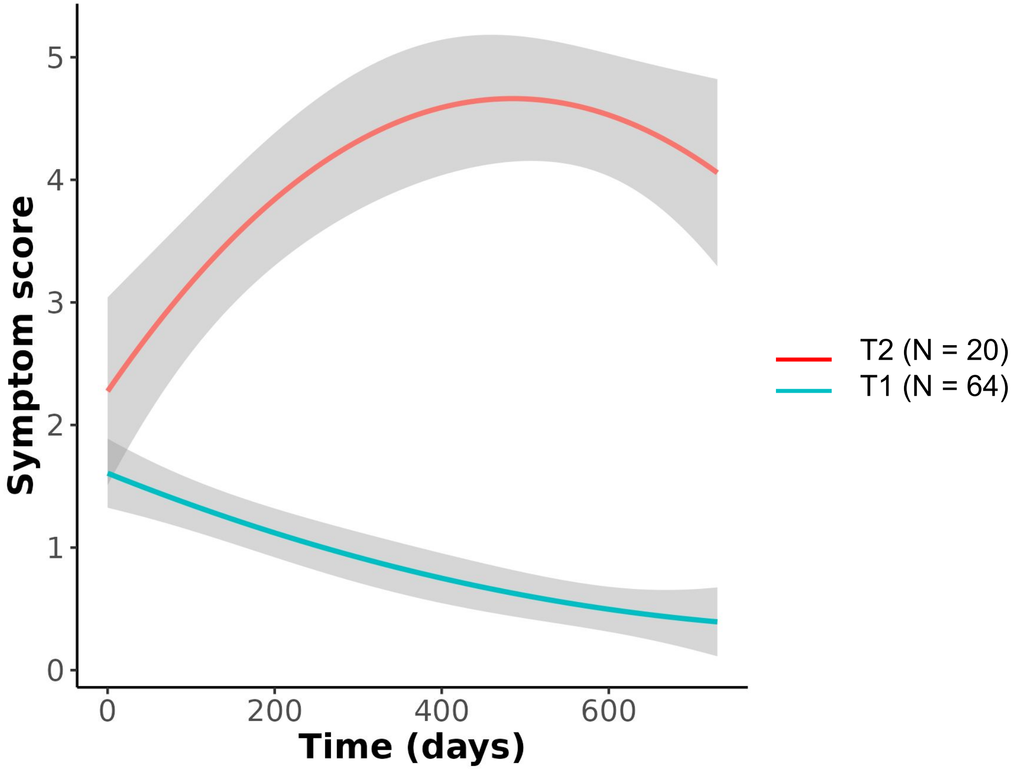

Supplementary figure 1: Complete case analysis: total symptom score evolution in T1 and T2 from baseline up to 24 months after (in days) for 84 participants who completed the 4 timepoints
